# Frequency and clinical implications of the isolation of rare nontuberculous mycobacteria

**DOI:** 10.1186/s12879-014-0741-7

**Published:** 2015-01-09

**Authors:** Junghyun Kim, Moon-Woo Seong, Eui-Chong Kim, Sung Koo Han, Jae-Joon Yim

**Affiliations:** Division of Pulmonary and Critical Care Medicine, Department of Internal Medicine, Seoul National University College of Medicine, 101 Daehak-Ro, Jongno-Gu, Seoul, 110-744 Republic of Korea; Department of Laboratory Medicine, Seoul National University College of Medicine, 101 Daehak-Ro, Jongno-Gu, Seoul, 110-744 Republic of Korea

**Keywords:** Nontuberculous mycobacteria, Clinical manifestation

## Abstract

**Background:**

To date, more than 125 species of nontuberculous mycobacteria (NTM) have been identified. In this study, we investigated the frequency and clinical implication of the rarely isolated NTM from respiratory specimens.

**Methods:**

Patients with NTM isolated from their respiratory specimens between July 1, 2010 and June 31, 2012 were screened for inclusion. Rare NTM were defined as those NTM not falling within the group of eight NTM species commonly identified at our institution: *Mycobacterium avium*, *M. intracellulare*, *M. abscessus*, *M. massiliense*, *M. fortuitum*, *M. kansasii*, *M. gordonae*, and *M. peregrinum*. Clinical, radiographic and microbiological data from patients with rare NTM were reviewed and analyzed.

**Results:**

During the study period, 73 rare NTM were isolated from the respiratory specimens of 68 patients. Among these, *M. conceptionense* was the most common (nine patients, 12.3%). The median age of the 68 patients with rare NTM was 68 years, while 39 of the patients were male. Rare NTM were isolated only once in majority of patient (64 patients, 94.1%). Among the four patients from whom rare NTM were isolated two or more times, only two showed radiographic aggravation caused by rare NTM during the follow-up period.

**Conclusions:**

Most of the rarely identified NTM species were isolated from respiratory specimens only once per patient, without concomitant clinical aggravation. Clinicians could therefore observe such patients closely without invasive work-ups or treatment, provided the patients do not have decreased host immunity towards mycobacteria.

## Background

Nontuberculous mycobacteria (NTM) are defined as mycobacteria other than *Mycobacterium tuberculosis* complex and *M. leprae*. Since the recognition of NTM as possible pathogens in the 1950s [1,2], the observed occurrence of NTM lung diseases has been increasing worldwide [3-5]. This may be attributed in part to improvements in microbial diagnostic tools leading to increased isolation of NTM. Additionally, the concomitant increase in susceptible hosts such as patients with underlying lung disease or an immunocompromised state may also contribute to the observed increase in NTM lung disease [5-9].

Presently, more than 125 classes of NTM species have been identified [10]. Furthermore, newly identified species of NTM are constantly reported owing to advances in technologies for the detection for NTM. For example, new species such as *M. fragae* and *M. paragordonae* were identified as recently as 2013 [11,12].

Although the clinical characteristics of diseases caused by commonly isolated NTM such as *M. avium*, *M. intracellulare* or *M. abscessus* are well known, those caused by newly recognized and rarely isolated NTM are not yet fully understood. In the present study, we investigated the frequency of rare NTM isolation and the clinical characteristics of patients with rare NTM.

## Methods

### Study population

Patients from whom NTM were isolated one or more times from respiratory specimens such as sputum, bronchoscopic wash fluid and bronchoalveolar lavage (BAL) fluid between July 1, 2010 and June 31, 2012 at Seoul National University Hospital, were included in the analysis. Hence, a total of 2556 NTM isolated from 1373 patients during the study period were analyzed retrospectively. The Institutional Review Board of Seoul National University Hospital approved the study protocol and waived the requirement for obtaining patient consent.

### Identification of NTM species

Respiratory specimens were decontaminated with 4% sodium hydroxide (NaOH), homogenized, and concentrated by centrifugation at 3000 × *g* for 20 min. The processed sediments were stained using the Ziehl-Neelsen method [13]. Concentrated specimens were cultured in MGIT tubes (Becton-Dickinson and Co.; Sparks, MD, USA) as well as in 3% Ogawa medium and observed weekly for 6 or 9 weeks after inoculation, respectively. Once cultured, *M. tuberculosis* and NTM were differentiated using the Gen-Probe® method (Gen-Probe; San Diego, CA, USA) [14]. Following isolation of a suspected mycobacterial species, confirmation of NTM was performed by analyzing the sequences of three genes: *16S rRNA*, *rpoB*, and *tuf*. Polymerase chain reaction (PCR) and subsequent sequence were performed and the resulting sequences were compared with those in the reference database using the basic local alignment search tool (BLAST). Mycobacterial species were identified using the *16S rRNA* sequences and the algorithm described in the Clinical and Laboratory Standards Institute guidelines MM18-A [15].

### Definition of rare NTM

For the purposes of this study, ‘rare NTM’ were defined as NTM species other than *M. avium*, *M. intracellulare*, *M. abscessus*, *M. massiliense*, *M. fortuitum*, *M. kansasii*, *M. gordonae and M. peregrinum*, which are the eight NTM species commonly identified at our institution.

### Clinical and radiographic characteristics

Demographic, clinical and radiographic data of the patients from whom rare NTM were isolated were reviewed. Demographic data including age, gender, and smoking habits; past medical history of tuberculosis (TB), measles, pertussis, and sinusitis; comorbidities including malignancy, diabetes mellitus, cerebrovascular disease, rheumatic disease, inflammatory bowel disease, gastroesophageal reflux disease, and underlying lung disease; clinical data for self-reported symptoms; and findings of the physical examinations, were all thoroughly reviewed. The characteristics and distribution of lung lesions were analyzed based on chest computed tomography (CT) by two pulmonologists (J.K. and J.J.Y.), who were aware of the patients’ NTM results. Patients from whom rare NTM were isolated more than once were analyzed separately.

## Results

### Common NTM isolated from respiratory specimens during the study period

During the study period, 2554 NTM were isolated from 1373 patients. Of these, 803 NTM were identified at the species level. Among these, 730 (90.9%) were commonly isolated NTM species while the others (73 specimens, 9.1%) were rare NTM. *M. avium* (250 specimens, 34.2%) and *M. intracellulare* (252 specimens, 34.5%) were the most frequently identified rare NTM species, followed by *M. abscessus* (93 specimens, 12.7%; see Table 1).

**Table 1:** **NTM commonly isolated from respiratory specimens during the study period**

**Species**	**N (%)**
*Mycobacterium avium complex*	
*Mycobacterium avium*	250 (34.2)
*Mycobacterium intracellulare*	252 (34.5)
*Mycobacterium abscessus complex*	
*Mycobacterium abscessus*	93 (12.7)
*Mycobacterium massiliense*	53 (7.3)
*Mycobacterium fortuitum*	28 (3.8)
*Mycobacterium gordonae*	19 (2.6)
*Mycobacterium kansasii*	18 (2.5)
*Mycobacterium peregrinum*	17 (2.4)
Total	730 (100.0)

### Rare NTM isolated during the study period

During the study period, 73 NTM isolated from 68 patients were identified as rare NTM. Of those isolated, 70 (95.9%) were from sputum and three (4.1%) were from bronchoscopic specimens such as bronchial washsing and bronchoalveolar lavage. Among the identified rare NTM, *M. conceptionense* was the most common (nine specimens, 12.3%), followed by *M. chelonae*, *M. lentiflavum* and *M. mageritense* (seven specimens each, 9.6% respectively; Table 2). Rare NTM were isolated only once from 64/68 patients, twice from three patients, and three times from one patient. Among the patients from whom rare NTM was isolated only once, commonly isolated NTM was also isolated 2.4 times from 8.2 samples of sputum on average.

**Table 2:** **Rare NTM species isolated from respiratory specimens during the study period**

**Species**	**N (%)**
*Mycobacterium conceptionense*	9 (12.3)
*Mycobacterium chelonae*	7 (9.6)
*Mycobacterium lentiflavum*	7 (9.6)
Mycobacterium mageritense*	7 (9.6)
*Mycobacterium chimaera*†	6 (8.2)
*Mycobacterium terrae*	5 (6.8)
*Mycobacterium kumamotonense*	4 (5.5)
*Mycobacterium porcinum*	3 (4.1)
*Mycobacterium goodie*	2 (2.7)
*Mycobacterium nebraskense*	2 (2.7)
Mycobacterium phocaicum*	2 (2.7)
*Mycobacterium septicum*	2 (2.7)
*Mycobacterium celatum*	1 (1.4)
*Mycobacterium holsaticum*	1 (1.4)
*Mycobacterium senuense*	1 (1.4)
*Mycobacterium arupense*	1 (1.4)
*Mycobacterium kubicae*	1 (1.4)
*Mycobacterium neoaurum*	1 (1.4)
*Mycobacterium xenopi*	1 (1.4)
Failed species identification‡	10 (13.7)
**Total**	73 (100.0)

*M. mageritense and *M. phocaicum* were isolated from a single patient.

†*M. chimaera* was isolated three times from a single patient.

‡Identification was attempted but failed at species level.

### Characteristics of the patient cohort from whom rare NTM were isolated

The median age of the 68 patients from whom rare NTM were isolated was 68 years (range, 30–84 years), while 39 (57.4%) were male. Of these patients, 24 (35.3%) had a previous history of TB treatment. The most common underlying diseases were solid organ malignancies including lung cancer (seven patients, 10.3%), and diabetes mellitus (six patients, 8.8%). TNF-α inhibitors were used in two patients and HIV infection was confirmed in one patient. Sputum production (39 patients, 57.4%) and coughing (30 patients, 44.1%) were the most common self-reported symptoms. Postnasal drip (10 patients, 14.7%) and crackles (four patients, 5.8%) were the most common findings during physical examination (Table 3).

**Table 3:** **Demographics and clinical characteristics of the patient cohort from whom rare NTM were isolated**

Demographics	N (%)
Age (year), median (range)	68.0 (30.0–84.0)
Sex (Male)	39/68 (57.4)
Body Mass Index (kg/m^2^), median (range)	22.4 (15.9–29.0)
Habitual factors	N (%)
Smoking	19/68 (33.8)
Ex-smoker	13/68 (22.4)
Current smoker	6/68 (10.3)
Past medical history	N (%)
Previous history of TB	24/68 (35.3)
Sinusitis	9/68 (13.2)
Measles	3/68 (4.4)
Pertussis	1/68 (1.5)
Comorbidities	N (%)
Malignancies	7/68 (10.3)
Lung cancer	1/68 (1.5)
Diabetes	6/68 (8.8)
Cerebrovascular disease	2/68 (2.9)
Rheumatoid arthritis	3/68 (4.4)
Inflammatory bowel disease	0/68 (−)
Gastroesophageal reflux	0/68 (−)
Underlying lung disease	
COPD	4/68 (5.9)
Asthma	4/68 (5.9)
TB-destroyed lung	3/68 (4.4)
Interstitial lung disease	1/68 (1.5)
Bronchiectasis	23/68 (33.8)
HIV infection	1/68 (1.5)
Post-transplantation status	0/68 (−)
Symptoms	N (%)
Cough	30/68 (44.1)
Dyspnea	13/68 (19.1)
Hemoptysis	11/68 (16.2)
Sputum	39/68 (57.4)
Fever	6/68 (8.8)
Myalgia	4/68 (5.9)
Weight loss	6/68 (8.8)
Physical examination	N (%)
Postnasal drip	10/68 (14.7)
Crackle	4/68 (5.8)
Wheezing	1/68 (1.5)
Murmur	0/68 (−)
Clubbing	0/68 (−)
Peripheral edema	0/68 (−)
Drug use	
TNF-alpha inhibitor^*^	1/68 (1.5)
Steroid (5–10 mg daily)^*^	3/68 (4.4)
Other immunomodulatory drugs^*^	2/68 (2.9)

^*^All these drugs were used for the treatment of patients with rheumatoid arthritis.

Upper lobes of lung were commonly involved (right upper lobe in 40 patients and left upper lobe in 33 patients). Bilateral lesions were found in 30 patients (44.1%). In 21 (30.9%) patients, three or more lobes were involved. Multiple nodules were the most commonly observed radiographic finding (42 patients, 61.8%). Bronchiectasis (24 patients, 35.3%) and cavities (13 patients, 19.1%) were also commonly observed in radiographic examinations (Table 4).

**Table 4:** **Radiographic findings in patient cohort with rare NTM**

	**N (%)**
Lesion location	
Right upper lobe	40/68 (58.8)
Right middle lobe	21/68 (30.9)
Right lower lobe	22/68 (32.4)
Left upper lobe	33/68 (48.5)
Left lower lobe	21/68 (30.9)
Lesion distribution	
Bilateral	30/68 (44.1)
Multilobar (≥3 lobes with abnormalities)	21/68 (30.9)
Lesion characteristics	
Multiple nodules	42/68 (61.8)
Bronchiectasis	24/68 (35.3)
Cavity	13/68 (19.1)
Unilateral	9/68 (13.2)
Bilateral	4/68 (5.9)
Radiographic classification	
Upper lobe cavitary pattern	12/68 (17.6)
Nodular bronchiectatic pattern	17/68 (25.0)
Unclassifiable	39/68 (57.4)

### Clinical course of four patients from whom rare NTM were isolated more than once

Four patients from whom rare NTM were isolated two or more times had follow-ups for a median duration of 378 days (range, 294–477 days). Initial chest CT showed patterns of bronchiectasis (patient 3), small nodules (patients 1, 3 and 4), consolidations (patients 2 and 4), and cavities (patient 4). In one patient (patient 2 in Table 5) from whom rare NTM as well as *M. abscessus* was isolated, the respiratory symptoms were aggravated and radiographic lesions progressed on follow-up chest CT. The clinician who managed this patient judged that this progression was caused by *M. abscessus* rather than *M. goodie*. Subsequently, a clarithromycin-based regimen was initiated for this patient. Negative conversion of sputum culture was not however achieved with treatment despite a slight symptomatic improvement. In the other two patients (patients 1 and 3 in Table 5), neither aggravation of symptoms nor progression of radiographic lesions on chest radiography occurred. These patients were observed without concomitant anti-NTM treatment. The other patient from whom *M. chimaera* was isolated at three separate time points (patient 4 in Table 5) developed new centrilobular nodules. However, this patient’s other symptoms did not change and no treatment was applied (Table 5).

**Table 5:** **Clinical characteristics of four patients from whom rare NTM were isolated more than once**

**Patients (sex/age)**	**Chief complaint**	**Underlying diseases**	**CT finding**	**Isolated NTM (number of times isolated)**	**Progression of radiographic lesions**	**Treatment (Y/N)**
1 (F/74)	Sputum	• History of tuberculous cervical lymphadenitis	• Nodules and subsegmental atelectasis	*M. conceptionense* (2)	• Not definite	No
2 (F/33)	Cough	• Undergoing jejunostomy (due to lye ingestion)	• Consolidations and branching opacities	*M. abscessus* (2)	• Increase in number of multiple centrilobular nodules and extent of bronchiectasis	Yes
	Sputum					
	Weight loss	• Bipolar I disorder		*M. goodii* (2)		
						Clarithromycin, Rifampin, Ethambutol, Moxifloxacin
						(Sep. 2007–Oct. 2009)
3 (F/63)	Bloody sputum	• Diabetes	• Bronchiectasis and nodules	*M. phocaicum* (1)	• Not definite	No
		• Coronary artery disease		*M. mageritense* (1)		
4 (M/59)	Cough	• History of pulmonary TB	• Nodules, consolidations, fluid-containing cavity	*M. chimaera* (3)	• New centrilobular nodules and patchy consolidation	No
	Hemoptysis					

## Discussion

New NTM are constantly reported, while the lung diseases arising from these new organisms are reported at a similarly rapid rate [16-19]. Consequently, clinicians inevitably encounter patients who present with unfamiliar and rarely identified NTM.

In the present study, various species of rarely identified NTM were isolated from respiratory specimens. *M. conceptionense* was the most frequently isolated rare NTM. This species was reported to cause infections of the skin and subcutaneous fat following surgical procedures in immunocompetent patients [20-22]. Moreover, another study demonstrated that this NTM may be a lung pathogen [18]. However, in our study, for the patient from whom *M. conceptionense* was isolated at two separate time points, neither aggravation of the symptoms nor progression of radiographic lesions was identified.

Other rare NTM species isolated from patients in the present study (*M. lentiflavum*, *M. mageritense*, *M. chimaera and M. xenopi*) may also cause lung diseases [16,18,19,23]. However, yet other rare NTM isolated from these patients, such as *M. kumamotonense* and *M. celatum*, have generally been considered to be misidentifications or a result of culture contamination, and are thus deemed to be clinically non-pathogenic organisms [24,25].

NTM classified as rarely isolated NTM in this study may prove common in other geographic regions. For example, *M. xenopi* is commonly identified in southern Ontario, South East England and Europe [26,27], but rarely in Australia, South America, USA and Asia [28,29]. However, in this study performed in South Korea, *M. xenopi* was isolated only once.

Despite their pathogenic potential, among the patients from whom rare NTM were identified, rare NTM were isolated only once in the majority of patients (64/68). Once-off isolation of the majority of the rare NTM suggests limited clinical significance of these NTM. Additionally, the observation that only one of the three patients from whom rare NTM were isolated two or more times without co-infection with common NTM showed evidence of radiographic aggravation, further substantiates the minimal clinical significance of rare NTM.

The limited clinical importance of the frequent one-time isolation of rare NTM presented in this study underscores the importance of the current ATS/IDSA diagnostic guidelines, which require repeated isolation of an NTM in the appropriate clinical setting [13]. Clinicians confronted with rare NTM could observe the patients for a while watching whether the same species of NTM would be isolated again or not.

To fully appreciate these results, a limitation of this study should be noted: species level identification was not performed on all NTM isolated from respiratory specimens. A prospective study including species identification of all NTM isolated during the certain period would likely confirm the findings of the current study.

## Conclusions

Rarely identified NTM isolated from respiratory specimens have limited clinical importance in most cases. Clinicians who treat patients with rarely identified NTM could therefore observe them closely without any intensive work-ups or treatment being required, provided that these patients do not present with decreased host immunity towards mycobacteria.

## Abbreviations

NTM: Nontuberculous mycobacteria
BAL: Bronchoalveolar lavage
TB: Tuberculosis
CT: Computed tomography
